# Determining the association of C-reactive-protein–triglyceride–glucose index and diabetes using machine learning and LASSO regression: A cross-sectional analysis of NHANES 2001 to 2010 results

**DOI:** 10.1097/MD.0000000000044815

**Published:** 2025-09-19

**Authors:** Lin Xi, Jijing Zhao, Yunpeng Wang

**Affiliations:** a The First People’s Hospital of Jiande, Hangzhou, China.

**Keywords:** CTI, diabetes, LASSO regression, machine learning, NHANES, nomogram, SHAP

## Abstract

The C-reactive protein–triglyceride–glucose index (CTI) has emerged as a novel metric for evaluating the severity of inflammation and the degree of insulin resistance. Nevertheless, the precise correlation between CTI and diabetes remains to be elucidated. Consequently, in this study, we elucidate the relationship between CTI and diabetes. The study utilized data from the National Health and Nutrition Examination Survey spanning from 2001 to 2010. To evaluate the association between CTI and the risk of diabetes, the research employed weighted logistic regression, subgroup analyses, and restricted cubic spline. Subsequently, participants were randomly assigned to the training and validation cohorts in a 7:3 ratio. Least Absolute Shrinkage and Selection Operator (LASSO) regression was employed to evaluate the validation cohort, select the optimal model, and identify potential confounding factors. The variables identified by LASSO regression were used to construct a nomogram-based predictive model, receiver operating characteristic curve, calibration curve, and decision curve analysis curve. The variables selected by LASSO regression were also incorporated into the ML model, and SHAP visualization analysis was performed. Upon adjustment for potential confounding factors, a significant positive correlation was observed between the CTI and the incidence of diabetes (OR = 1.96, 95% CI: 1.69–2.26, *P* < .001). Restricted cubic spline showed a linear positive correlation between CTI and incidence of diabetes mellitus (*P*-nonlinear = .5200). A total of 8 variables were identified through LASSO regression, including age, race, marital status, hypertension, body mass index, cardiovascular disease (CVD), and CTI. A nomogram-based predictive model was constructed using these predictors. The area under the receiver operating characteristic curve (AUC) in the validation cohort was 0.92, indicating a robust performance of the model. These 8 variables were subsequently incorporated into the ML model, and the CatBoost model demonstrated the best performance with an AUC of 0.843 (95% CI: 0.820–0.866). SHAP analysis revealed that hypertension was the most influential factor. A significant positive linear correlation was observed between higher CTI values and increased diabetes risk, suggesting that CTI has the potential to serve as a predictor for the incidence risk of diabetes.

## 1. Introduction

Diabetes has become a major public health problem globally, and its incidence continues to rise, seriously affecting the health and quality of life of the population. Over the past few decades, the prevalence of diabetes has significantly increased in both developed and developing countries, making it a significant global health priority.^[1]^ A systematic analysis for the Global Burden of Disease Study indicates that in 2021, there were 529 million people with diabetes globally, with a higher prevalence in males than females, resulting in a male-to-female ratio of 1:14, and it is projected that the number of people with diabetes will exceed 1.31 billion by 2050.^[2]^ According to the research by Ke-Jie He et al, the mortality rate among people with diabetes is lowest in China, Japan, South Korea, Nordic countries (Sweden, Norway, Finland), and Canada (<54.09 per 100,000 people). In contrast, the highest mortality rates are observed in South Africa, Egypt, Oman, and Mexico, ranging from 255.81 to 1176.73 per 100,000 people.^[3]^

C-reactive protein (CRP) is an acute time-phase reactive protein that is usually significantly elevated in conditions such as inflammation and infection. Recent evidence has highlighted a significant correlation between CRP levels and the development and progression of diabetes, particularly in individuals with type 2 diabetes mellitus (T2DM).^[4,5]^ CRP, as a marker of inflammation, is significantly elevated in patients with T2DM and is closely associated with metabolic disorders such as insulin resistance (IR) and hyperlipidemia.^[6–8]^

IR – defined as reduced insulin sensitivity in liver, skeletal muscle and adipose tissue – is a central pathophysiological driver of T2DM.^[9]^ The triglyceride–glucose (TyG) index, calculated from fasting triglyceride and glucose concentrations, has been validated as a simple and reliable surrogate marker of IR.^[10]^ Studies have shown that elevated TyG values are associated with a higher risk of developing diabetes.^[11–13]^

In 2022, Ruan et al first proposed the CTI to comprehensively assess inflammation and IR status.^[14]^ CTI was established on CRP and TyG indices. Some studies have shown that CTI is important in predicting cancer mortality,^[15]^ endometriosis,^[16]^ erectile dysfunction,^[17]^ depressive symptoms,^[18]^ coronary heart disease,^[19]^ stroke,^[20]^ etc. Moreover, CTI is derived from laboratory tests, which has the advantages of being inexpensive and easy to obtain. However, the correlation between CTI and diabetes has not been adequately investigated. Given the background information on the association between CRP levels and diabetes, especially T2DM, the primary objective of this study was to examine the relationship between the CTI and diabetes mellitus. This was achieved through a large-sample, comprehensive, population-based cross-sectional study design.

Typically, traditional predictive models rely on high-quality, structured data. Yet, the presence of numerous feature variables in high-dimensional data often leads to overfitting, prolonged model training times, and diminished predictive accuracy. ML algorithms, as a subset of artificial intelligence, offer a solution by effectively handling complex nonlinear relationships in high-dimensional data. They enhance the accuracy of information extraction from big data and simplify the analysis of raw data. ML has been extensively applied in the medical field for disease prediction. SHAP visualization analysis is a leading method for interpreting ML decision-making processes. It provides an intuitive quantification of each feature’s contribution to model predictions and circumvents the limitations of traditional “black box” models.

## 2. Methods

### 2.1. Data sources

The data utilized in this study were derived from the National Health and Nutrition Examination Survey (NHANES). The study protocol of NHANES was approved by the Research Ethics Review Board of the National Center for Health Statistics, and informed consent was obtained from all participants. This study was designed as a cross-sectional analysis of publicly released NHANES 2001 to 2010 data.

### 2.2. Study population

**Figure 1.**
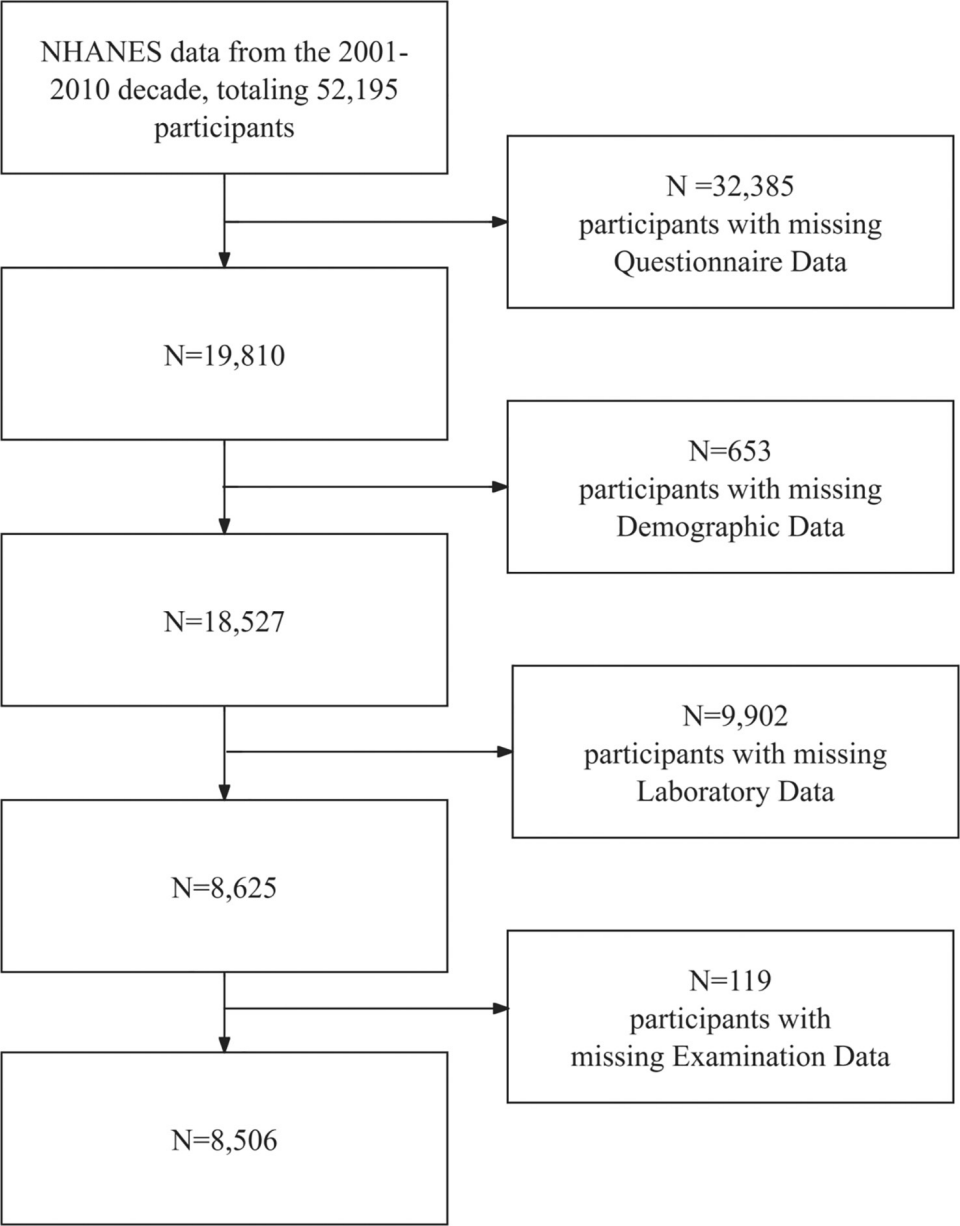
Flowchart of participant selection from the NHANES 2001 to 2010. NHANES = National Health and Nutrition Examination Survey.

This study included 52,195 participants in NHANES from 2001 to 2010. Exclusion criteria included 32,385 participants with missing questionnaire data, 653 participants with missing demographics data, 9902 participants with missing laboratory data, and 119 participants with missing examination data (Fig. 1).

### 2.3. Definitions of CTI and diabetes

The calculation formula for the CTI is as follows: CTI = 0.412 × Ln (C-reactive protein) (mg/dL) + Ln [triglycerides (mg/dL) × fasting blood glucose (mg/dL)/2]. The exposure variable in our study is CTI. Fasting plasma glucose was obtained after an overnight fast (≥8 hours) and measured by the hexokinase method (NHANES laboratory protocol). We restricted the analysis to NHANES 2001 to 2010 because CRP was only measured in participants during the 1999 to 2010 cycles. To ensure complete laboratory data for calculating the CTI (which requires both CRP and fasting plasma glucose/triglyceride values), later cycles without CRP measurements could not be included.

Diabetes diagnosis was ascertained through a self-report questionnaire (DIQ010, DIQ050, DIQ070), which included the following question: “Other than during pregnancy, have you ever been told by a doctor or health professional that you have diabetes or sugar diabetes?” “Are you now taking insulin?” “Are you now taking diabetic pills to lower your blood sugar? These are sometimes called oral agents or oral hypoglycemic agents.” Participants had 2 options to choose from for their answer: “Yes” or “No.”

### 2.4. Study variables

Covariates included demographics data (age, gender, race, education level, poverty-to-income ratio, and marital status), examination data (body mass index (BMI)), and questionnaire data (smoking habits, frequency of alcohol consumption, hypertension, cancer, and CVD). The diagnosis of hypertension was based on 2 questions: “Were you told on 2 or more different visits that you had hypertension, also called high blood pressure (self-report questionnaire BPQ030)?” (2) “Because of your high blood pressure, have you ever been told to take prescribed medicine? (self-report questionnaire BPQ040A)” Smoking habits were categorized based on the answers to 2 questions: “Have you smoked at least 100 cigarettes in your entire life? (self-report questionnaire SMQ020)” and “Do you now smoke cigarettes? (self-report questionnaire SMQ040)” CVD includes a range of diseases, including stroke (self-report questionnaire MCQ160f), angina (self-report questionnaire MCQ160d), congestive heart failure (self-report questionnaire MCQ160b) and coronary artery disease (self-report questionnaire MCQ160c).

### 2.5. Statistical analysis

Clinical data were analyzed using R 4.4.2 software. In this study, weighted logistic regression analysis was conducted to investigate the relationship between CTI and the prevalence of diabetes. The study analyzed the data through 3 stepwise-adjusted models: model 1 presents unadjusted raw values; model 2 builds on model 1 by incorporating covariates such covariates as demographic factors, BMI, smoking habits, and frequency of alcohol consumption; and model 3 further builds on model 2 by incorporating variables related to chronic diseases such as hypertension, CVD, and cancer. To investigate whether there was a nonlinear or linear relationship between CTI and diabetes, the study used the restricted cubic spline (RCS) for the analyses, testing 3 nodes while controlling for other potential confounders. The study also used subgroup analyses to illustrate the stability of the association between CTI and diabetes, which included multiple variables such as age, gender, race, education, marital status, BMI, smoking status, CVD, hypertension, and cancer.

To address the challenges posed by high-dimensional data and multicollinearity among variables, we employed least absolute shrinkage and selection operator (LASSO) regression. LASSO is a penalized regression method that simultaneously performs variable selection and regularization, which is particularly suitable for datasets with a large number of predictors relative to the number of observations. This approach helps to mitigate the risk of overfitting and enhances the interpretability of the model by shrinking less important predictor coefficients to zero. The LASSO regression model was fit to the training dataset, and the selected variables were subsequently used to construct a predictive model. The performance of the model was evaluated using the validation dataset, with metrics such as the AUC and calibration curves employed to assess the model’s predictive accuracy and calibration. Additionally, we performed decision curve analysis (DCA) to evaluate the clinical utility of the model.

Given the complexity and high dimensionality of the data, which often pose challenges to the performance of ML algorithms, we employed LASSO regression for feature selection prior to constructing the ML models. This approach allowed us to identify the most critical predictors for outcome prediction while reducing the dimensionality of the dataset and mitigating the risk of overfitting. The selected variables were subsequently incorporated into the ML analyses.

In medical data analysis, the integration of ML with traditional statistics has become a growing trend. Traditional statistical methods provide a robust theoretical foundation and intuitive interpretability, whereas ML excels in handling large datasets and automating complex analyses. Within the mlr3 framework, we constructed a suite of discriminative models, including logistic regression, support vector machine, gradient boosting machine (GBM), neural network, random forest, Xgboost, K-nearest neighbors (KNN), adaboost, light GBM (LightGBM), and CatBoost.

Benchmarking is a crucial method for systematically evaluating and comparing the performance of ML models. The AUC was used as the primary metric for model selection, while additional metrics provided supplementary assessments of model performance. To minimize evaluation bias in the ML models, we employed 10-fold cross-validation for data resampling. Differences in performance metrics among the models were assessed using analysis of variance and Kruskal-Wallis H tests.

To evaluate the overall feature importance in the ML model with the best predictive performance, we utilized SHAP values. SHAP represents a recent advancement in enhancing the interpretability of tree-based models by employing a game-theoretic approach to aggregate local contributions of individual features, thereby explaining the model’s behavior on a global scale. This method is considered superior to other global approximation techniques, as it not only quantifies the importance of features within the model but also provides insights into the role of each feature in specific predictions.

## 3. Results

### 3.1. Characteristics of participants

In this study, after excluding participants with missing data, we included a total of 8506 participants, including 888 patients with diabetes (Fig. 1). Compared with nondiabetic patients, men, older individuals, those with increased alcohol consumption, higher BMI, elevated CTI, former smokers, and patients with hypertension, CVD, and cancer have a higher risk of developing diabetes (Table 1).

**Table 1:** Demographic baseline chart of NHANES Participants from 2001 to 2010.

Characteristics	Overall (N = 8506)	Nondiabetes (N = 7618)	Diabetes (N = 888)	*P*-value
Gender				
Male	4517 (52%)	3996 (52%)	521 (57%)	.046
Female	3989 (48%)	3622 (48%)	367 (43%)	
Age	46.08 ± (16.05)	45.17 ± (15.84)	57.60 ± (14.00)	<.001
Race				
Mexican American	1587 (7.7%)	1394 (7.6%)	193 (8.2%)	<.001
Other Hispanic	519 (3.9%)	453 (3.9%)	66 (4.3%)	
Non-Hispanic White	4585 (74%)	4203 (75%)	382 (66%)	
Non-Hispanic Black	1530 (9.7%)	1310 (9.2%)	220 (15%)	
Other Race – including multi-racial	285 (4.4%)	258 (4.2%)	27 (5.9%)	
Education level				
<9th grade	932 (5.3%)	770 (4.9%)	162 (11%)	<.001
9–11th grade	1320 (12%)	1140 (11%)	180 (16%)	
High school graduate/GED or equivalent	2017 (25%)	1811 (25%)	206 (26%)	
Some college or AA degree	2428 (31%)	2206 (31%)	222 (30%)	
College graduate or above	1809 (27%)	1691 (28%)	118 (17%)	
Marital status				
Married	4705 (58%)	4194 (58%)	511 (61%)	<.001
Spouse deceased	643 (4.9%)	528 (4.5%)	115 (10%)	
Divorced	902 (10%)	780 (10%)	122 (14%)	
Separated	263 (2.4%)	229 (2.3%)	34 (3.2%)	
Never married	1308 (16%)	1240 (16%)	68 (7.0%)	
Living with partner	685 (8.1%)	647 (8.4%)	38 (4.7%)	
Ratio of family	3.15 ± (1.60)	3.18 ± (1.60)	2.82 ± (1.55)	<.001
BMI (kg/m^2^)	28.36 ± (6.37)	28.03 ± (6.15)	32.65 ± (7.40)	<.001
Smoke				
Never	3936 (47%)	3574 (47%)	362 (40%)	<.001
Former	2495 (28%)	2148 (27%)	347 (37%)	
Current smoker	2075 (25%)	1896 (26%)	179 (23%)	
Alcohol frequency	4.61 ± (21.21)	4.65 ± (20.89)	4.15 ± (24.91)	<.001
Hypertension				
Yes	2568 (26%)	1969 (23%)	599 (65%)	<.001
No	5938 (74%)	5649 (77%)	289 (35%)	
CVD				
Yes	807 (7.2%)	573 (5.8%)	234 (24%)	<.001
No	7699 (93%)	7045 (94%)	654 (76%)	
Cancer				
Yes	782 (8.7%)	651 (8.2%)	131 (15%)	<.001
No	7724 (91%)	6967 (92%)	757 (85%)	
CTI	7.97 ± (0.93)	7.91 ± (0.89)	8.73 ± (1.04)	<.001

### 3.2. Association of CTI with the prevalence of diabetes

**Figure 2.**
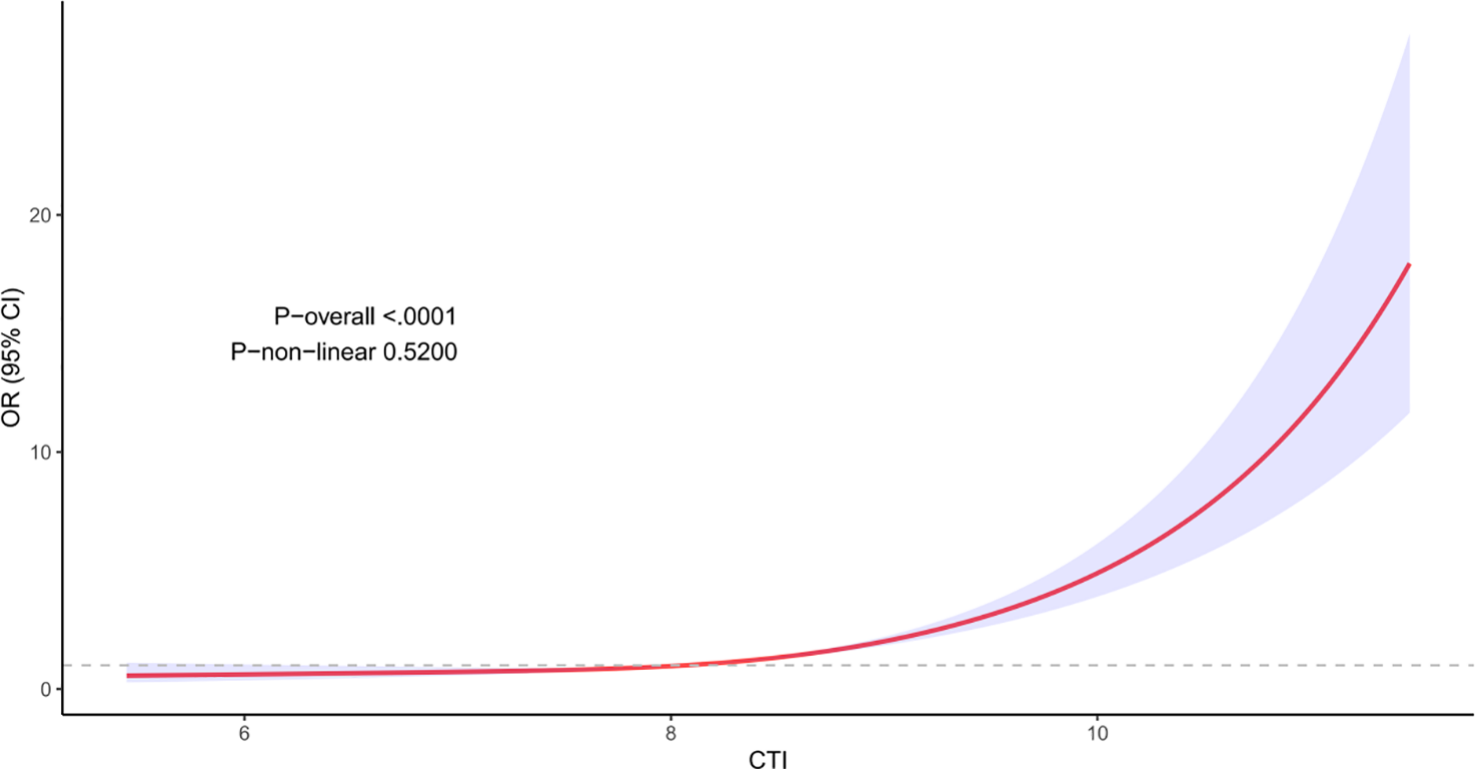
Restricted cubic spline analysis demonstrating a linear association between the C-reactive protein–triglyceride–glucose index and the prevalence of diabetes (*P*-nonlinear = .5200).

CTI was divided into quartiles and used to assess its association with diabetes prevalence (Table 2). We found that CTI showed a significant positive association with the prevalence of diabetes in all 3 different models, and all were statistically significant (*P* < .001). Furthermore, after adjusting for all covariates (Model 3), the OR and its corresponding 95% CI for the highest versus the lowest quartile of CTI were as follows: OR = 3.23 ([2.13–4.90], *P* for trend <.01), for every 1-unit increase in CTI, there was an increase in the probability of prevalence of diabetes by 223%. In addition, RCS curve analysis showed a significant linear correlation between CTI and the prevalence of diabetes (*P*-nonlinear = .5200) (Fig. 2).

**Table 2:** Multivariate regression of C-reactive protein–triglyceride–glucose index with diabetes.

	Model 1 OR (95% CI)	Model 2 OR (95% CI)	Model 3 OR (95% CI)
CTI	2.56 (2.26–2.89)	2.04 (1.77–2.34)	1.96 (1.69–2.26)
Q1	Reference	Reference	Reference
Q2	2.22 (1.46–3.37)	1.25 (0.83–1.89)	1.27 (0.83–1.94)
Q3	3.61 (2.34–5.57)	1.61 (1.04–2.47)	1.59 (1.03–2.46)
Q4	9.04 (6.13–13.3)	3.40 (2.29–5.06)	3.23 (2.13–4.90)
*P*-value for trend	<.001	<.001	<.001

BMI = body mass index, CI = confidence interval, CTI = C-reactive protein–triglyceride–glucose index, CVD = cardiovascular disease, OR = odds ratio, PIR = poverty-to-income ratio.

Model 2: adjusts for ethnicity, age, gender, PIR, BMI, educational attainment, marriage condition, alcohol frequency, and smoking behavior.

Model 3: adds hypertension, CVD, and cancer to the adjustments in Model 2.

### 3.3. Subgroup analyses

**Figure 3.**
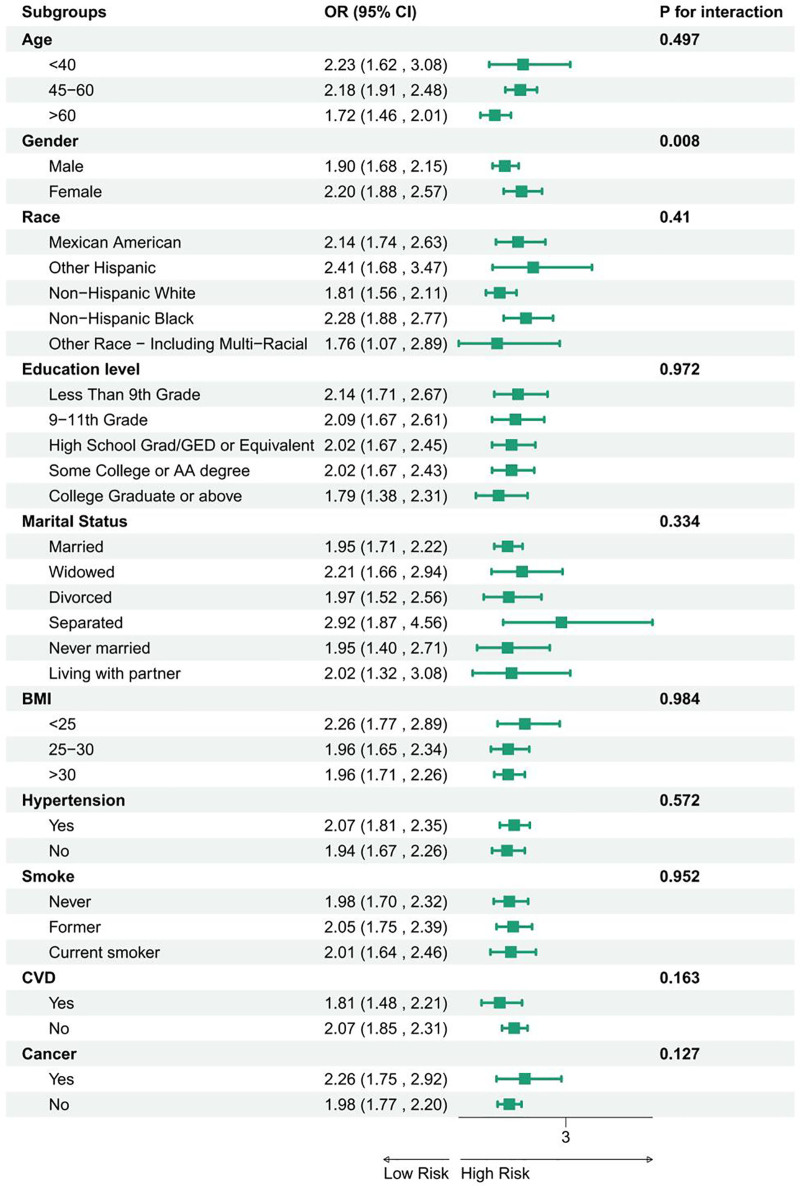
Subgroup analyses of the association between the CTI and diabetes prevalence across age, sex, race, education, marital status, BMI, hypertension, CVD, smoking, and cancer. BMI = body mass index, CTI = C-reactive protein–triglyceride–glucose index, CVD = cardiovascular disease.

The effect of different population characteristics on the relationship between CTI and diabetes prevalence was further explored in subgroup analyses. The analysis found that CTI and the prevalence of diabetes remained stable in the subgroups of age, race, education level, marital status, BMI, hypertension, CVD, smoking, and cancer (*P* for interaction >.05) (Fig. 3).

In the present study, 4517 cases (52%) were males and 3989 cases (48%) were females. *P* for interaction for gender was <.05, indicating a significant interaction of gender on the relationship between CTI and diabetes.

### 3.4. LASSO regression analysis and construction of the nomogram prediction model

**Figure 4.**
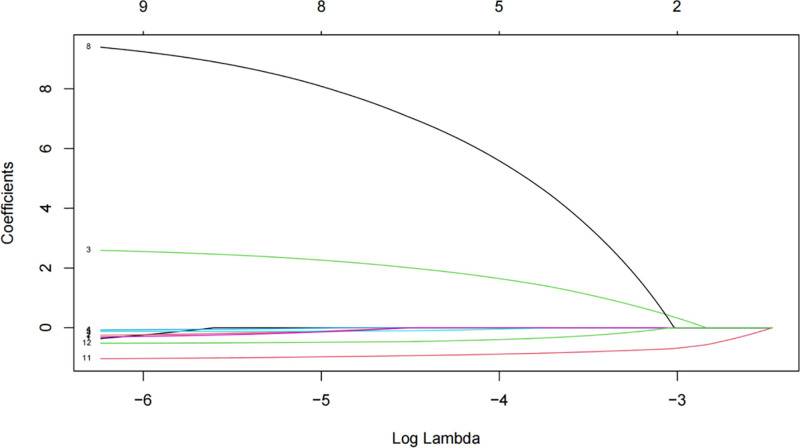
LASSO path plot illustrating coefficient shrinkage of predictors as the penalty parameter (λ) increases. LASSO = least absolute shrinkage and selection operator.

Eight variables were selected through LASSO regression analysis, including age, race, marital status, hypertension, BMI, CVD, and CTI. In this study, we utilized LASSO regression to perform feature selection. The LASSO path plot (Fig. 4) visually demonstrates the selection process of the predictors. As the penalty parameter (λ) increases, the coefficients of less important predictors shrink to zero, while those of more important predictors remain non-zero across a broader range of λ values. This plot provides a clear indication of which predictors are most relevant for the outcome of interest.

**Figure 5.**
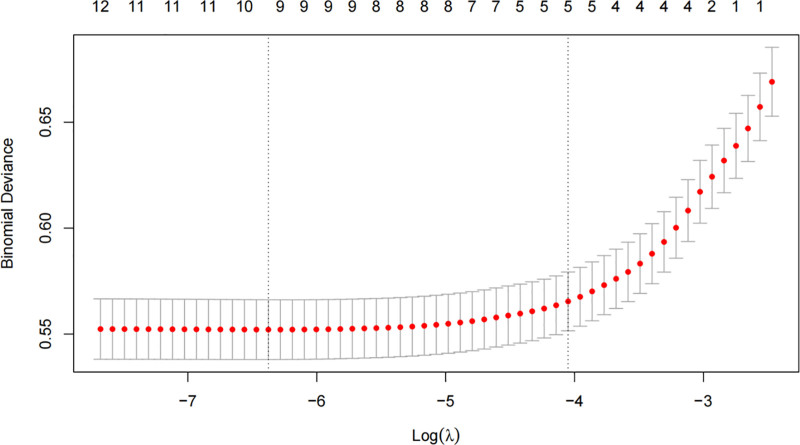
Ten-fold cross-validation plot for selecting the optimal λ value in the LASSO regression model based on minimum mean cross-validated error.

The cross-validation error plot (Fig. 5) was used to determine the optimal value of λ. The plot displays the mean cross-validation error against the logarithm of λ, with the minimum error indicating the optimal λ. This optimal λ was selected to balance model complexity and predictive performance, ensuring that the LASSO regression model achieved the best trade-off between bias and variance. The selected predictors based on this optimal λ were subsequently used in the subsequent analyses.

**Figure 6.**
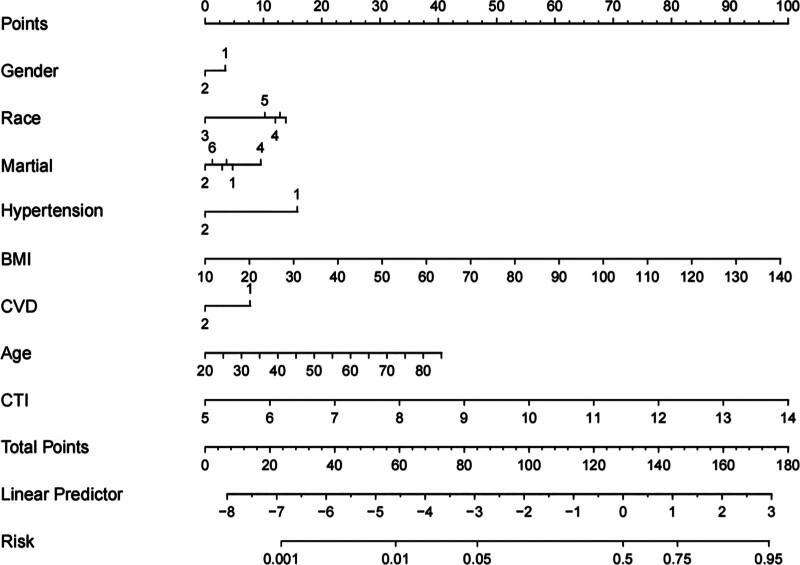
Nomogram for predicting the risk of diabetes constructed from variables selected by LASSO regression: age, race, marital status, hypertension, BMI, CVD, sex, and the CTI. Hypertension and CVD: 1, yes; 2, no. martial status: 1, married; 2, spouse deceased; 3, divorced; 4, separated; 5, never married; 6, living with partner. Gender: 1, Male; 2, Female. Race: 1, Mexican American; 2, Other Hispanic; 3, Non-Hispanic White; 4, Non-Hispanic Black; 5, Other Race-Including Multi-Racial. BMI = body mass index, CTI = C-reactive protein–triglyceride–glucose index, CVD = cardiovascular disease, LASSO regression model based on minimum mean cross-validated error.

**Figure 7.**
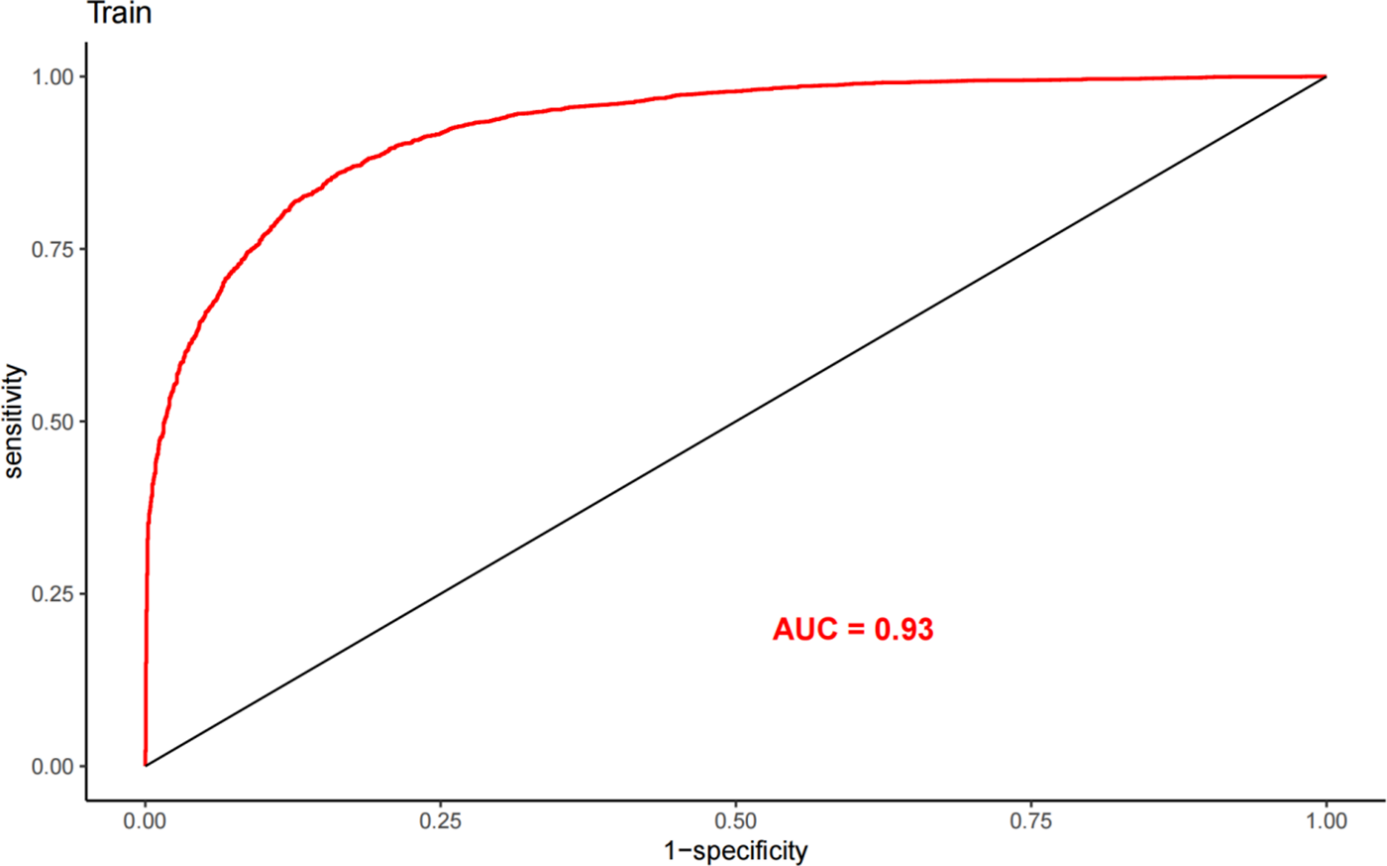
ROC curve of the nomogram in the training cohort (area under the curve = 0.93). ROC = receiver operating characteristic.

**Figure 8.**
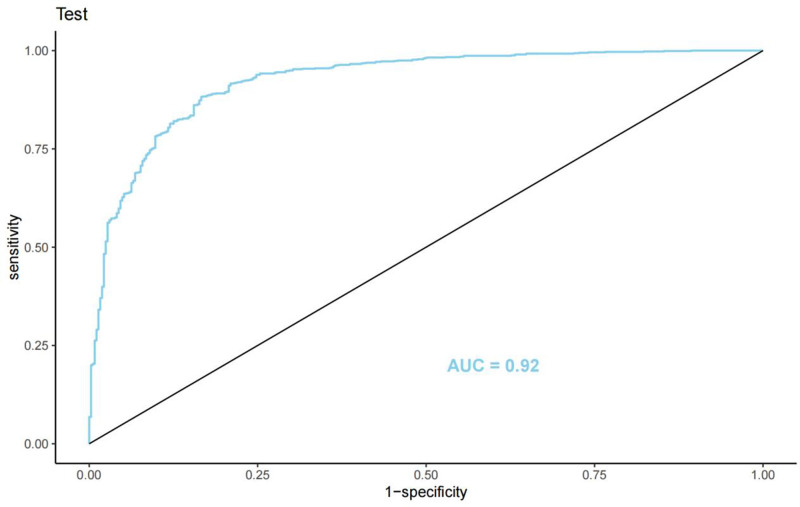
ROC curve of the nomogram in the validation cohort (area under the curve = 0.92). ROC = receiver operating characteristic.

**Figure 9.**
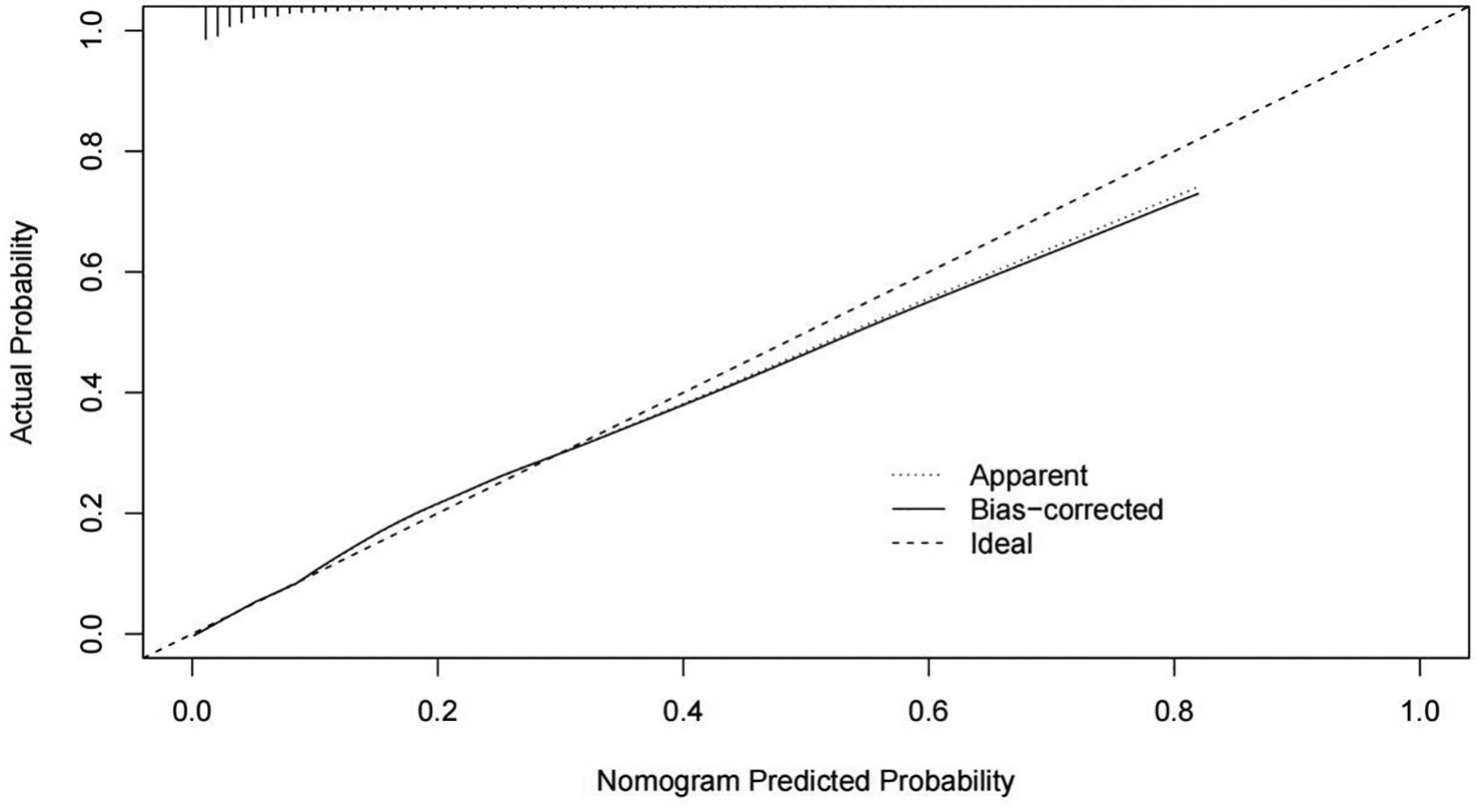
Calibration plot of the nomogram comparing predicted versus observed probabilities of diabetes in the validation cohort.

**Figure 10.**
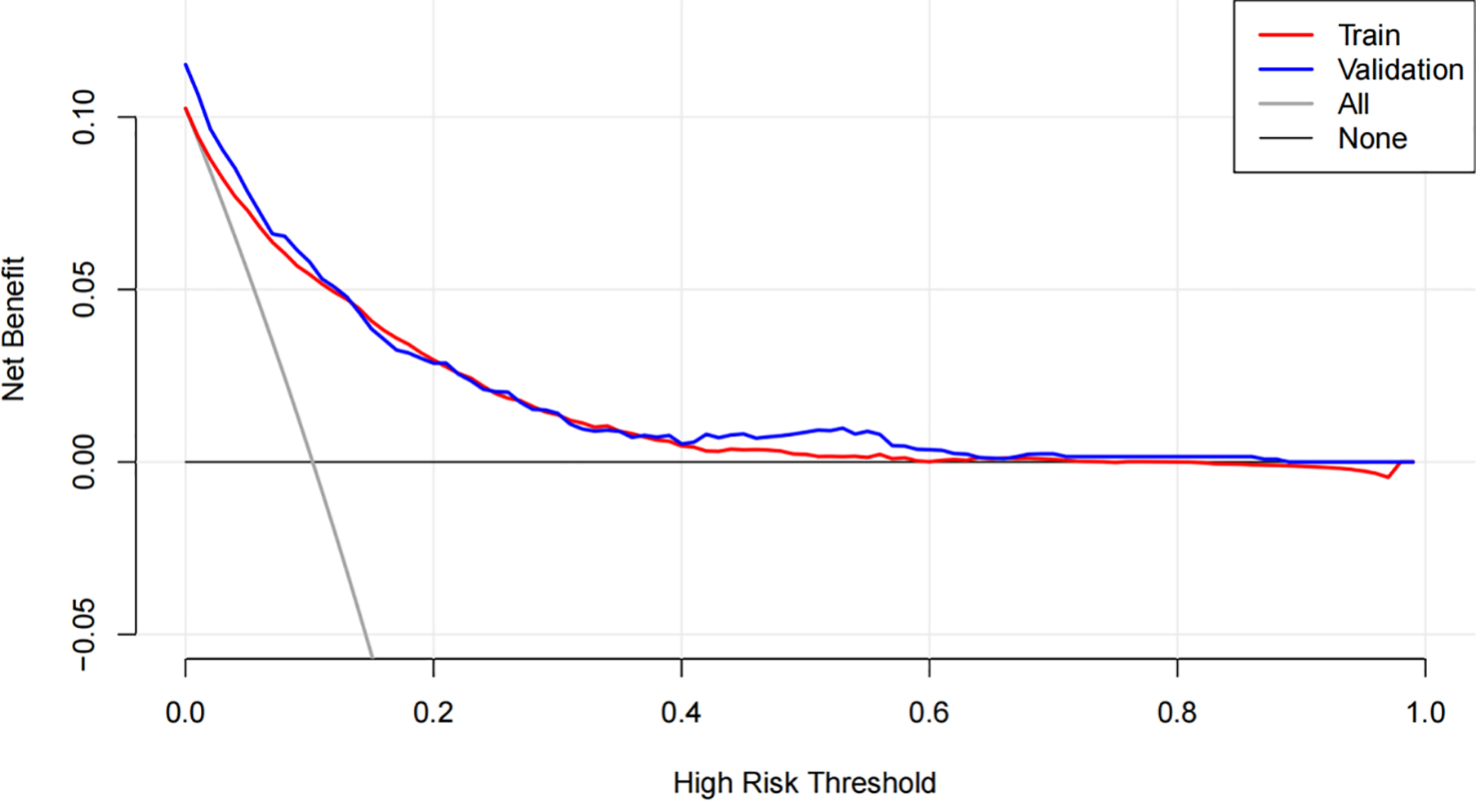
Decision curve analysis evaluating the clinical net benefit of the nomogram across a range of threshold probabilities.

The LASSO regression model demonstrated a satisfactory predictive ability in the validation cohort, with an AUC of 0.92. Based on the variables selected by LASSO regression, a nomogram-based predictive model was constructed (Fig. 6). Additionally, receiver operating characteristic curves (Figs. 7 and 8), calibration curves (Fig. 9), and DCA curves (Fig. 10) were generated to comprehensively evaluate the performance of this model.

### 3.5. Feature selection in ML model development and validation

**Figure 11.**
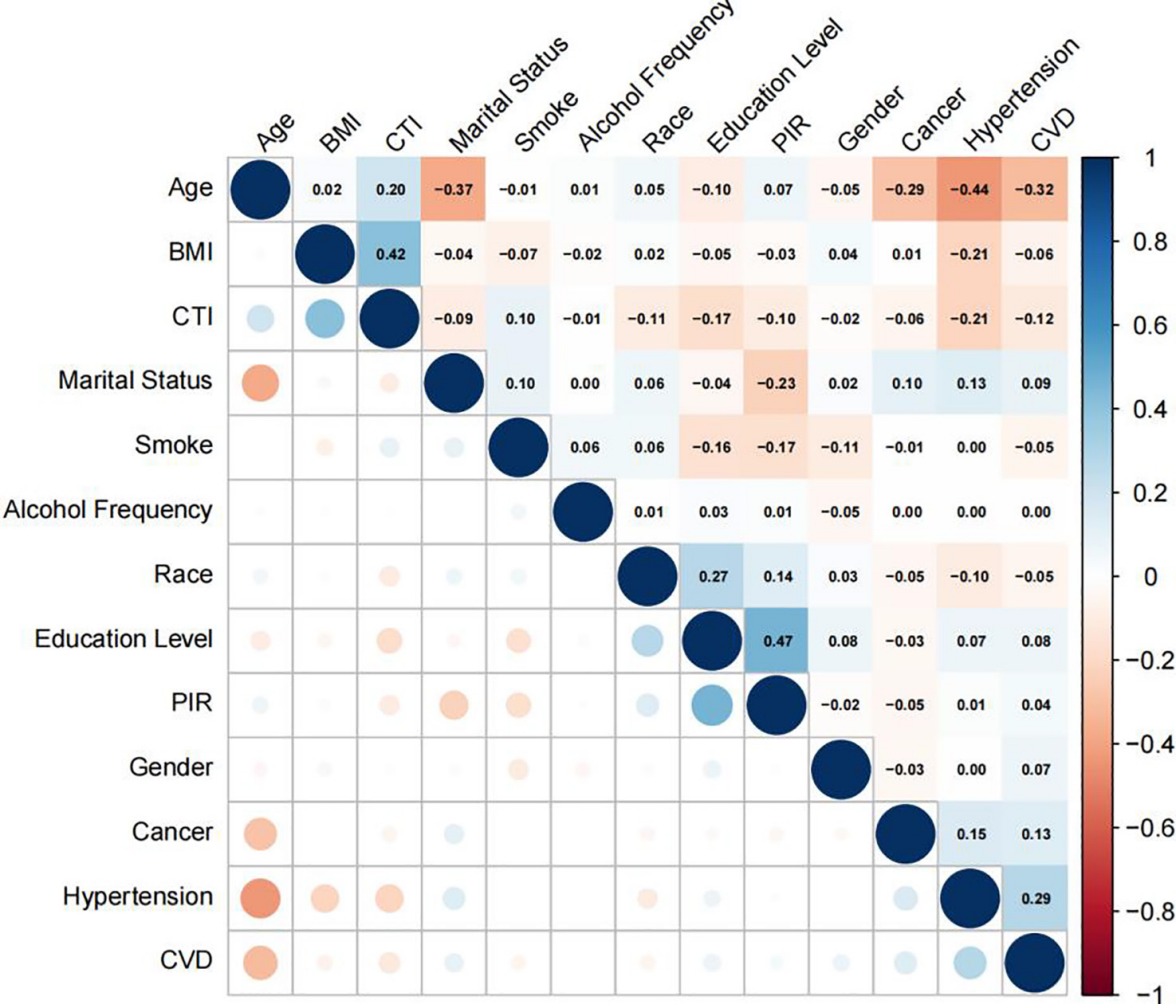
Correlation heatmap of study variables.

The correlation matrix for the study variables is presented in Figure 11, and the significant relationships are highlighted. In the present study, age was found to be significantly negatively correlated with hypertension, the poverty-to-income ratio was significantly positively correlated with educational level.

In the performance evaluation of various ML models for predicting the diabetes, distinct differences were observed across key metrics such as accuracy, sensitivity, specificity, precision, and F1 score (Table 3).

**Table 3:** Assessment of performance metrics for 10 mL models.

Model	Accuracy	Sensitivity	Specificity	Precision	F1
Logistic	0.745	0.789	0.74	0.261	0.393
SVM	0.843	0.274	0.909	0.26	0.267
GBM	0.733	0.805	0.724	0.254	0.386
NeuralNetwork	0.764	0.759	0.764	0.273	0.401
RandomForest	0.772	0.665	0.785	0.265	0.379
Xgboost	0.734	0.816	0.724	0.256	0.39
KNN	0.854	0.289	0.92	0.296	0.293
Adaboost	0.784	0.586	0.807	0.261	0.361
LightGBM	0.73	0.741	0.728	0.241	0.363
CatBoost	0.76	0.767	0.76	0.271	0.4

GBM = gradient boosting machine, KNN = K-nearest neighbors, SVM = support vector machine.

**Figure 12.**
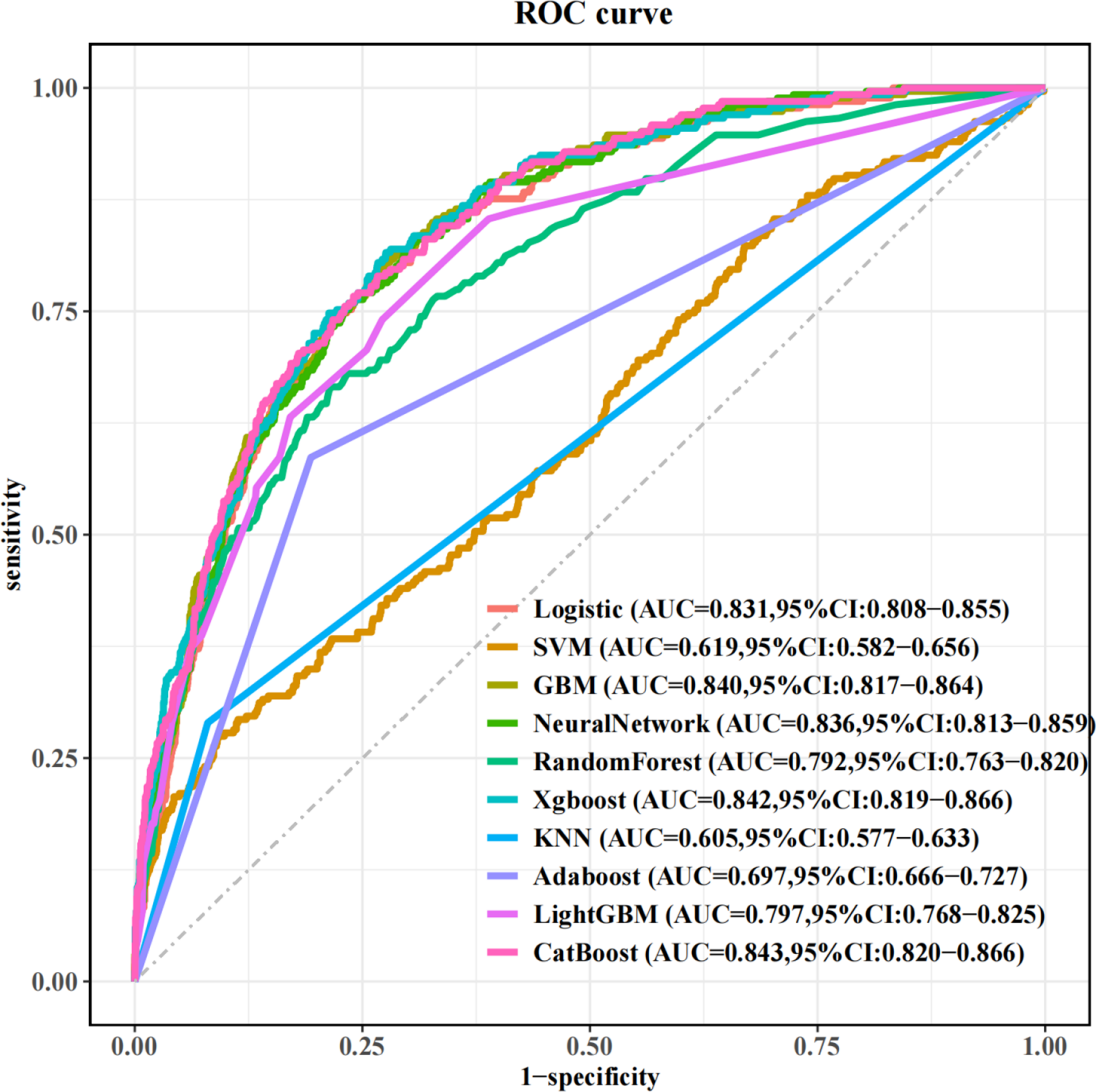
ROC curves comparing the performance of 10 machine-learning models in the validation cohort. ROC = receiver operating characteristic.

**Figure 13.**
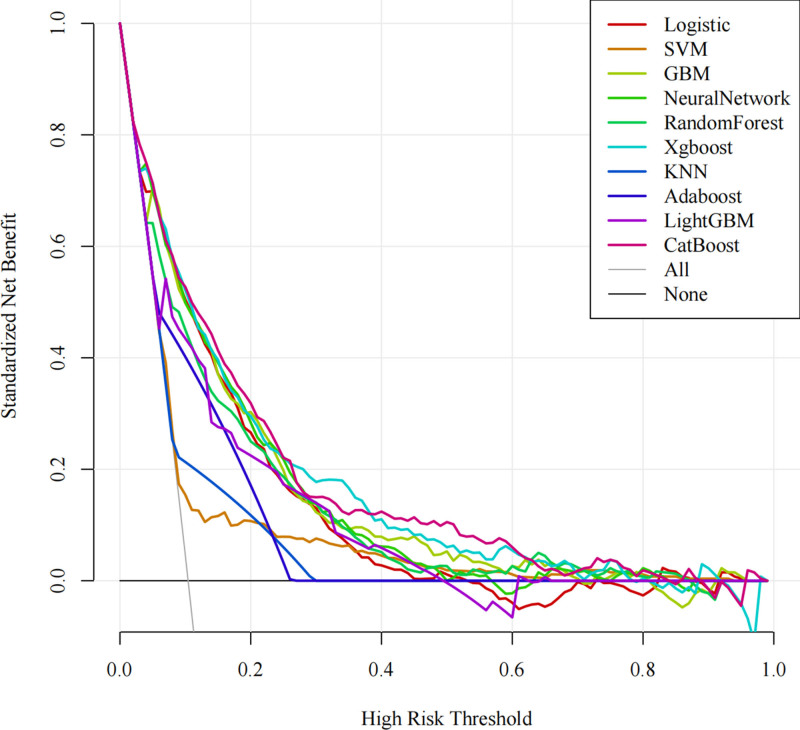
DCA curves comparing the clinical utility of 10 machine-learning models in the validation cohort. DCA = decision curve analysis.

During the evaluation of ML models, the AUC serves as a key indicator of a model’s capacity to differentiate between positive and negative cases. An AUC value closer to 1 signifies exceptional classification performance. In our analysis, the CatBoost model achieved the highest AUC value, demonstrating its superior ability to rank positive instances above negative ones. Although other metrics such as accuracy, specificity, or precision were not necessarily the highest (Table 3, Fig. 12), the CatBoost model was deemed the most appropriate for this study due to its overall performance. DCA was employed to compare the clinical utility of various predictive models (Fig. 13).

### 3.6. SHAP value interpretation

**Figure 14.**
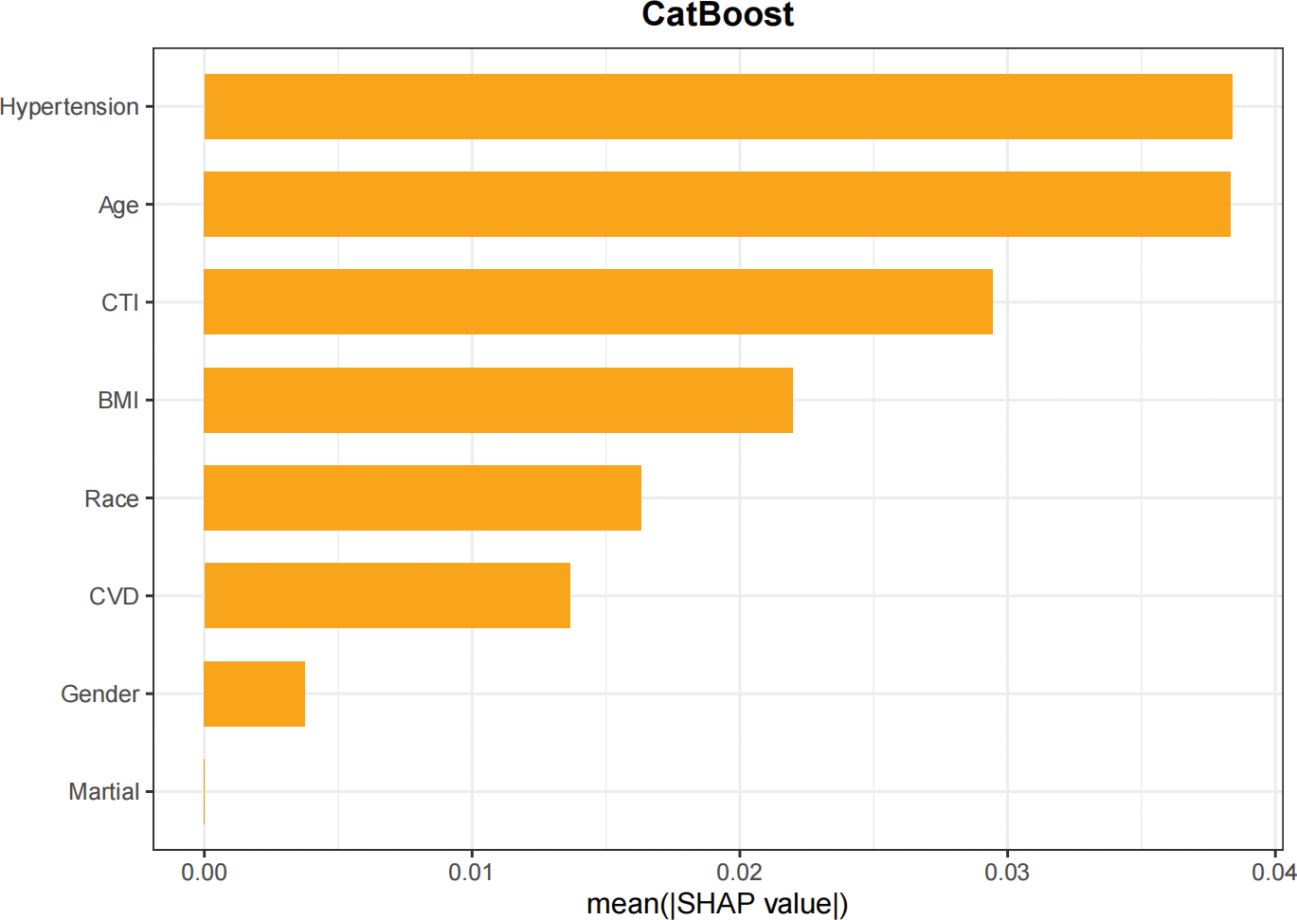
Bar plot of mean SHAP values ranking the importance of predictors in the CatBoost model.

**Figure 15.**
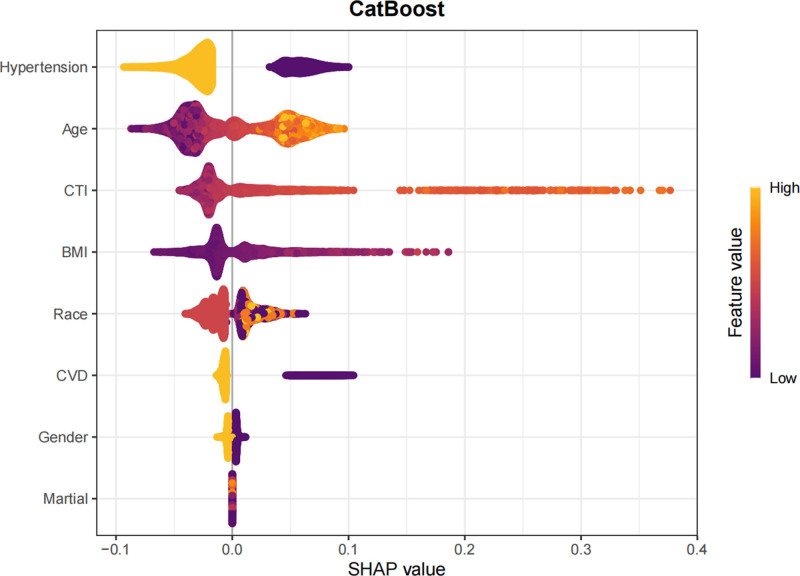
Beeswarm plot of SHAP values illustrating the distribution and impact of each predictor across individual participants.

**Figure 16.**
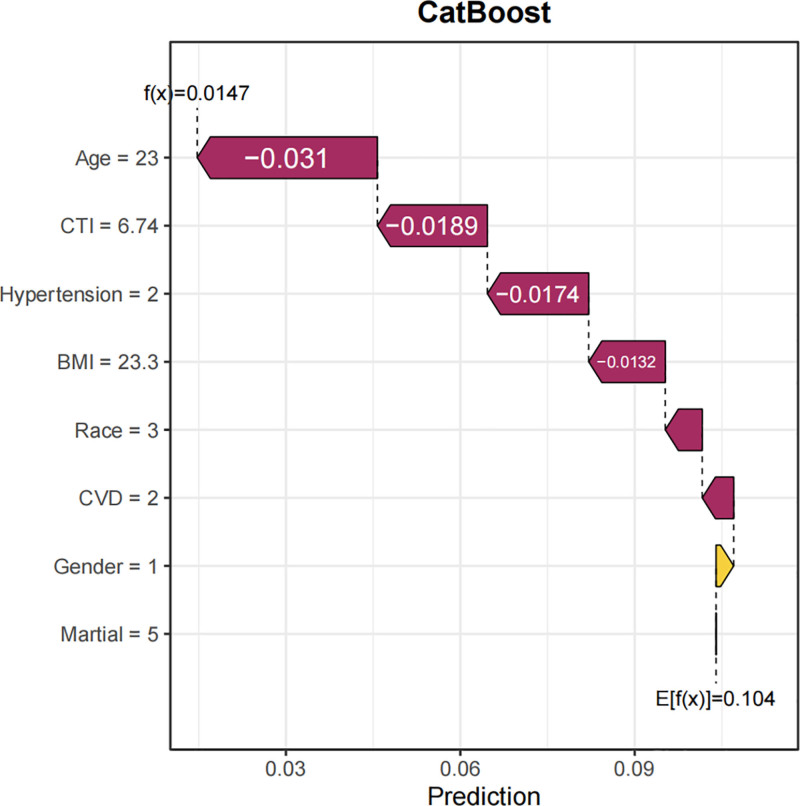
Waterfall plot of SHAP values for a representative participant showing individual contributions of predictors to the final diabetes probability.

**Figure 17.**
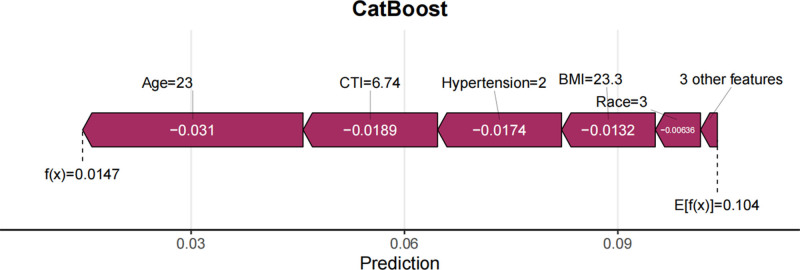
Force plot of SHAP values for a representative participant demonstrating the cumulative effect of predictors on the predicted diabetes risk.

In this study, SHAP analysis was utilized to visually interpret the contributions of feature variables to predictions within the optimal model, CatBoost. Specifically, a bar plot (Fig. 14) depicted the importance of feature variables and their mean SHAP values, with features ordered from most to least important. The beeswarm plot (Fig. 15) provided a global overview of feature distribution, sorted by mean SHAP values. Each point in this plot represented the SHAP value of a specific feature within an individual sample, with colors indicating the magnitude of the feature value (red for high values and blue for low values). This visualization clarified the relationships between feature values and their impact on predictions, including both monotonic and nonlinear patterns. Among the features, hypertension was identified as the most important predictor, with the highest mean SHAP value, followed by age, CTI, BMI, race, CVD, gender, and marital status. Waterfall plots (Fig. 16) and force plots (Fig. 17) further elucidated the contributions of feature variables to individual predictions. The waterfall plot provided a clear visualization of the ranking and magnitude of contributions from different features to the prediction of diabetes in each individual, with yellow arrows indicating positive contributions and brown arrows representing negative contributions. The force plot, in turn, presented the contributions of feature variables in an alternative format, with arrow colors and lengths representing the direction and magnitude of contributions, respectively, ultimately yielding the predicted output value for each individual.

## 4. Discussion

To our knowledge, this study represents the first investigation into the association between the CTI and the incidence of diabetes. Utilizing data from the NHANES, we employed weighted multifactorial logistic regression analysis, RCS, and subgroup analyses to explore this relationship. The analysis based on NHANES data from 2000 to 2010 revealed a significant positive association between CTI and diabetes incidence, with the risk of diabetes increasing progressively across CTI quartiles (Q1-Q4). This positive correlation remained consistent when CTI was categorized into quartiles. Subgroup analyses indicated that the association between CTI and diabetes prevalence was stable across various demographic and clinical subgroups, including age, race, education level, marital status, BMI, hypertension, cardiovascular disease, smoking status, and cancer history. However, a significant interaction was observed between gender and diabetes (*P* for interaction < .05), suggesting that the effect of CTI on diabetes risk may be modified by gender. The following reasons may exist: first, women were, on average, older than men at the time of T2DM diagnosis and had higher BMI and cholesterol levels.^[21,22]^ This suggests that gender may modify the relationship between CTI and diabetes by influencing metabolic profiles. Second, women have a more significant increased risk of CVD after developing T2DM.^[21]^ This suggests that gender may modify the relationship between CTI and diabetes by affecting cardiovascular health. In the general population, women have a lower risk of CVD due to the protective effect of estrogen, but in patients with T2DM, this protective effect disappears and women have an increased risk of CVD. This may explain why gender affects the relationship between CTI and diabetes.

IR is one of the core mechanisms in the pathogenesis of diabetes, and the TyG index has been widely validated as an effective alternative index for assessing IR. Studies by Zhao et al,^[11]^ Zhang et al,^[12]^ and Zhang et al^[13]^ found that TyG index was associated with an increased risk of diabetes and mortality. However, the traditional TyG index has limitations and fails to adequately consider the effect of the degree of inflammation on metabolism. Both CRP and TyG indices are strongly associated with diabetes. Given the established connections between inflammation and IR with diabetes, it is plausible to hypothesize that CTI may be associated with diabetes. Our findings support this hypothesis, demonstrating a significant relationship between CTI and diabetes incidence. As a novel index, CTI offers several practical advantages: it is easily obtainable and cost-effective. These attributes suggest that CTI holds promise as a predictive tool for identifying individuals at risk for developing diabetes.

In this study, we utilized LASSO regression to identify a set of clinically relevant predictors for diabetes, and subsequently constructed a nomogram-based predictive model incorporating these variables. Through LASSO regression, 8 variables were identified as significant predictors, including age, race, marital status, hypertension, BMI, CVD, and CTI (*P* < .05). The nomogram-based predictive model demonstrated robust performance, with an AUC of 0.92 in the validation cohort. This suggests that the model has strong discriminative ability and potential clinical utility for predicting diabetes. LASSO regression is a powerful statistical method for variable selection and regularization, particularly in scenarios where the number of potential predictors is large relative to the number of observations. By imposing a penalty on the absolute size of the regression coefficients, LASSO can shrink some coefficients to zero, effectively performing variable selection. In our study, LASSO regression identified 8 key variables from a larger pool of potential predictors, highlighting the most relevant factors associated with diabetes. This approach not only enhances the interpretability of the model but also reduces the risk of overfitting, which is a common concern in high-dimensional data analysis.

The nomogram-based predictive model constructed using the selected predictors provides a visual and quantitative tool for estimating the probability of diabetes. Nomograms are widely used in medical research due to their simplicity, interpretability, and ease of use in clinical settings. The high AUC value of 0.92 in the validation cohort indicates that the model has excellent discriminative ability, suggesting that it can effectively distinguish between individuals at high and low risk for diabetes. This level of performance is comparable to or better than many existing prediction models for similar outcomes, highlighting the potential clinical utility of our model.

The robust performance of the nomogram-based model can be attributed to several factors. First, the use of LASSO regression for variable selection ensured that only the most relevant predictors were included in the model, reducing the risk of overfitting and improving generalizability. Second, the combination of demographic, clinical, and imaging-based predictors allowed for a comprehensive assessment of risk, capturing multiple dimensions of the underlying pathophysiology. Finally, the rigorous validation process, including the use of an independent validation cohort, provided strong evidence of the model’s reliability and accuracy.

In this study, we employed a comprehensive ML approach to investigate the relationship between CTI and the risk of diabetes. Our findings revealed a positive correlation between CTI and the incidence of diabetes, suggesting that CTI may serve as a potential biomarker for diabetes risk assessment. Among the ten ML models utilized, the CatBoost model demonstrated the highest AUC value of 0.843. This superior performance indicates that CatBoost may be particularly well-suited for capturing the complex relationships between the various clinical features and the risk of diabetes.

The superior performance of the CatBoost model in this study can be attributed to several factors. CatBoost is designed to handle categorical features effectively, which is particularly advantageous in medical datasets that often contain a mix of numerical and categorical variables. Additionally, CatBoost’s implementation of gradient boosting with a focus on reducing overfitting and improving generalizability likely contributed to its robust performance in our analysis. The high AUC value of 0.843 suggests that CatBoost may be a valuable tool for developing accurate and reliable diabetes risk prediction models in clinical practice.

The SHAP visualization analysis provided valuable insights into the relative importance of various factors in predicting diabetes risk. Hypertension emerged as the most significant factor, consistent with the well-established epidemiological and clinical evidence linking hypertension to diabetes. The second most important factor was age, which is a well-known risk factor for diabetes due to age-related declines in insulin sensitivity and beta-cell function. CTI, BMI, race, CVD, gender, and marital status were also identified as important contributors to diabetes risk. These findings underscore the multifactorial nature of diabetes and highlight the importance of considering a comprehensive set of clinical, demographic, and imaging-based features in risk prediction models.

However, our study has several limitations that should be acknowledged. First, the dataset used for model training and validation was derived from a specific population, which may limit the generalizability of our findings to other populations with different demographic or clinical characteristics. Future studies should aim to validate our findings in diverse cohorts to ensure the robustness of the identified relationships and the predictive models. Second, while SHAP analysis provided insights into feature importance, it does not fully elucidate the complex interactions between features. Further investigation using techniques such as partial dependence plots or interaction analysis may help uncover more nuanced relationships. Third, while the nomogram provides a visual representation of the relative importance of each predictor, it does not fully capture the complex interactions between variables. Fourth, diabetes status was solely ascertained by self-reported physician diagnosis and medication use, without confirmatory laboratory data (e.g., fasting plasma glucose, HbA1c or 2-hour oral glucose tolerance test). Consequently, undiagnosed or misreported cases might have introduced misclassification bias, potentially attenuating the observed association between CTI and diabetes. Future studies should incorporate objective glycaemic measures to validate our findings. Additionally, hypertension, smoking status and cardiovascular disease were defined solely through self-reported questionnaire responses, which may be subject to recall or social-desirability bias and could lead to misclassification of exposures. Future studies should incorporate objective measurements (e.g., blood pressure readings, cotinine levels or clinical records) to validate these variables.

## 5. Conclusions

In this study, the correlation between CTI and diabetes was investigated based on NHANES large-sample data. The results showed that CTI was significantly and positively correlated with the prevalence of diabetes, and CTI can be used as a potential indicator for predicting the risk of diabetes and providing a reference for early prevention and intervention of diabetes. In this study, we employed a comprehensive approach combining LASSO regression and machine-learning techniques to investigate the relationship between CTI and diabetes incidence, and to develop a robust predictive model for diabetes risk. A nomogram-based predictive model incorporating these variables demonstrated excellent discriminative ability. Additionally, among various machine-learning models, CatBoost exhibited the highest performance.

BMI = body mass index, CTI = C-reactive protein–triglyceride–glucose index, CVD = cardiovascular disease, NHANES = National Health and Nutrition Examination Survey.

## Acknowledgements

Relevant data were obtained from the NHANES database. The authors thank the patients who contributed to this study. The authors extend their gratitude to all contributing investigators for their invaluable data contributions.

## Author contributions

**Conceptualization:** Lin Xi, Jijing Zhao, Yunpeng Wang.

**Data curation:** Lin Xi, Jijing Zhao, Yunpeng Wang.

**Formal analysis:** Lin Xi, Jijing Zhao, Yunpeng Wang.

**Funding acquisition:** Lin Xi, Jijing Zhao, Yunpeng Wang.

**Investigation:** Lin Xi, Jijing Zhao, Yunpeng Wang.

**Methodology:** Lin Xi, Jijing Zhao, Yunpeng Wang.

**Project administration:** Lin Xi, Jijing Zhao, Yunpeng Wang.

**Resources:** Lin Xi, Jijing Zhao, Yunpeng Wang.

**Software:** Lin Xi, Jijing Zhao, Yunpeng Wang.

**Supervision:** Lin Xi, Jijing Zhao, Yunpeng Wang.

**Validation:** Lin Xi, Jijing Zhao, Yunpeng Wang.

**Visualization:** Lin Xi, Jijing Zhao, Yunpeng Wang.

**Writing – original draft:** Lin Xi, Jijing Zhao, Yunpeng Wang.

**Writing – review & editing:** Lin Xi, Jijing Zhao, Yunpeng Wang.
